# Enhanced gut microbiome supplementation of essential amino acids in *Diploptera punctata* fed low-protein plant-based diet

**DOI:** 10.3389/finsc.2024.1396984

**Published:** 2024-04-22

**Authors:** Paul A. Ayayee, Nick Petersen, Jennifer Riusch, Claudia Rauter, Thomas Larsen

**Affiliations:** ^1^ Department of Biology, University of Nebraska Omaha, Omaha, NE, United States; ^2^ Department of Entomology, Insectary, BioSci Greenhouse, Ohio State University, Columbus, OH, United States; ^3^ Department of Archeology, Max Planck Institute of Geoanthropology, Jena, Germany

**Keywords:** *Diploptera punctata*, stable isotope, EAA provisioning, standard metabolic rate, gut microbiome

## Abstract

**Introduction:**

Building on our previous work, we investigate how dietary shifts affect gut microbial essential amino acid (EAA) provisioning in the lactating cockroach *Diploptera punctata*.

**Method:**

To that end, we fed cockroaches three distinct diets: a plant-only Gari diet composed of starchy and granulated root tuber Yucca (*Manihot esculenta*), a dog food diet (DF), and a cellulose-amended dog food (CADF) diet. We anticipated that the high carbohydrate, low protein Gari would highlight increased microbial EAA supplementation to the host.

**Results:**

By day 28, we observed distinct profiles of 14 bacterial families in the insect gut microbiomes of the three dietary groups. CADF-fed insects predominantly harbored cellulolytic and nitrogen-fixing bacteria families *Streptococcaceae and Xanthomonadaceae*. In contrast, Gari-fed insects were enriched in anaerobic lignocellulolytic bacteria families *Paludibacteraceae* and *Dysgonomonadaceae*, while DF-fed insects had a prevalence of proteolytic anaerobes *Williamwhitmaniaceae* and sulfate-reducing bacteria *Desulfovibrionaceae*. Furthermore, we confirmed significantly higher EAA supplementation in Gari-fed insects than in non-Gari-fed insects based on δ^13^C-EAA offsets between insect and their diets. The δ^13^C-EAA offsets between DF and CADF were nearly indistinguishable, highlighting the relevance of using the plant-based Gari in this experiment to unequivocally demonstrate this function in this insect. These results were underscored by lower standard metabolic rate (SMR) relative to the DF insect in Gari-fed (intermediate SMR and dietary quality) and CADF (least SMR and dietary quality) insects.

**Discussion:**

The influence of diet on EAA provisioning and SMR responses in insects underscores the need for further exploration into the role of gut microbial functions in modulating metabolic responses

## Introduction

Essential amino acid (EAA) provisioning by insect gut microbes supplements the host’s biological requirements or, in some instances, provides all the biological EAA requirements. However, a significant challenge in investigating gut microbial functions in insects lies in identifying the origins of EAAs. To address this issue, one strategy involves comparing the natural abundance of stable carbon stable isotope values in EAAs (δ^13^C_EAAs_) between insects and their diets, while observing some caveats. The first caveat is that only the domains, bacteria, fungi, and plants, can synthesize the full complement of EAAs and that synthesized EAAs have distinct δ^13^C_EAAs_ that enable the distinction of the origin of EAAs from bacteria, fungi, and plants. The second caveat is that for all consumers, consumer δ^13^C_EAAs_ will equal dietary δ^13^C_EAAs_ because animals generally incorporate EAAs directly from the diet without change (1–4). When this second caveat is not met, it indicates the presence of an alternate intrinsic source(s) of EAAs for the consumer. Following this, the source of alternative EAAs in insects can be inferred with source diagnostic δ^13^C_EAA_ patterns of bacteria, fungi, and plants (5). This approach has been used to determine alternate EAA contribution in a variety of macroinvertebrates (6–9), as well as insects (10–13).

Interpreting δ^13^C_EAA_ results for specialists feeding on single-sourced protein-deprived diets, e.g., wood-feeding beetles (11) and termites (10) or peat-feeding enchytraeid worms (14), tends to be straightforward. However, for omnivorous insects with mixed diets δ^13^C_EAA_, such as cockroaches (12, 13), or those on artificial diets (containing multiple sources of proteins) as the lab-reared beetle *Anoplophora glabripennis* (11), interpreting δ^13^C_EAA_ results becomes more complicated. A recent study determined for the first time that the viviparous Pacific beetle cockroach, *Diploptera punctata*, had mixed microbial (bacterial and fungal) EAA supplementation (13). However, identifying the specific contributions and biosynthetic origins of these supplemented δ^13^C_EAAs_ proved challenging. This complexity arises primarily from two issues: The composite nature of their diets (dog food, DF, and cellulose-amended dog food, CADF) and the predominance of fungal protein sources within these diets. These factors cloud the clarity of the EAAs’ origins and may limit the predictive ability of the models.

Elucidating insect gut microbiota function is crucial because it directly links gut microbial EAA provisioning to host metabolic phenotypes, often measured as standard metabolic rate (SMR) responses to external and internal cues. Low-quality diets not only decrease the body mass of insects but also alter their SMR across various insect orders (13, 15, 16). This relationship highlights how nutritional quality influences insect physiology and energy management, affecting their growth, health, and survival. Interpreting changes in SMR in ectotherms in response to dietary changes (low or high quality) is complex; in some species, SMR increases in response to dietary quality, and in other species, SMR decreases (17–20). While the direct effects of diet on SMR are often evident, the emerging understanding that diet composition significantly shapes the associated gut microbiome composition and its functions related to nutrition, measured as body mass (13, 21, 22), suggests indirect dietary impacts. This suggests that the dietary influence extends beyond immediate nutritional content, mediating effects on the host’s body mass through the gut microbiome, which directly connects to insect SMR. The study posits that dietary conditions alter microbe-mediated nutrient provisioning, which in turn affects the SMR of insect hosts. The premise underscores the complex interplay between diet, gut microbiome, and metabolic rate, suggesting that microbial nutrient provisioning is critical in mediating host metabolic responses to dietary changes​.

To clarify the role of gut microbial EAA provisioning in an omnivorous insect, this study sidesteps the complexities and masking effect associated with composite diets in previous studies by introducing a non-composite, entirely plant-based diet alongside the previously used DF and CADF diets (13). We address the challenge of confounding δ^13^C_EAA_ signals in composite diets. The DF diet (Nestlé Purina PetCare Company, MO, USA) is protein-rich with moderate carbohydrate and fat content. The CADF diet, a diluted DF diet with cellulose, is neither a phagostimulant nor a deterrent (23). The plant-based Gari diet is high in carbohydrates but low in fats and protein. Gari is a common food consumed in sub-Sahara Africa with low nutritional value. It is estimated to be compositionally made up of carbohydrates (33.6%), crude fiber (0.483%), protein (0.019%), fat (~0%), and ash (0.066%) (24). It also has a substantial amount of lignin and hemicellulose (25). Incorporating the new plant-based diet alongside the previously used CADF and DF diets allowed us to build upon the findings from 13, offering a direct baseline comparison to highlight gut EAA provisioning further. We hypothesized that there would be differences in the composition of the gut microbiome and increased microbial EAA provisioning (without the masking effects of mixed-protein sources) in insects fed the plant-only diet relative to the different composite diets. This will show an increased reliance on microbial EAA provisioning to compensate for dietary protein deficiencies. We also hypothesized that insects fed with CADF would increase SMR compared with insects fed the DF diet, as previously reported (13). Insects fed the plant-only diet are anticipated to have intermediate SMR as the plant diet is not expected to be resistant to digestion and impact the host’s physiology like the CADF diet.

## Materials and methods

### Insect rearing and experiment conditions

We used *D. punctata* based on availability and as a follow-up to our previous study (13). We maintained individuals (females) on three diets for 28 days. Females were chosen for this study, as starting point for further studies investigating resource allocation in this viviparous species under similar dietary regimes. We assessed potential diet-induced changes in the gut microbiome and microbial EAA provisioning functions of the microbiome at the end of the feeding period and investigated gut microbiome-mediated changes in mass-specific standard metabolic rate (SMR) on days 1 and 28. *Diploptera punctata* cockroaches used in the colony were obtained from the Evolution, Ecology, and Organismal Biology department and the Insectary and BioSci Greenhouse at The Ohio State University in early 2022. Cockroaches were maintained in ventilated plastic containers, provided with water *ad libitum* weekly, and fed pulverized dog food (Nestle-Purina, St. Louis, MO). Cockroaches were maintained at 28 °C and a relative humidity of 40%. The individuals used in the study were all females from the main colony.

### Measurement of carbon dioxide production and standard metabolic rate calculation

Thirty females maintained on a dog food diet in the main colony were selected and assigned to three different diets: an optimal composite dog food diet (high-quality diet, DF), a sub-optimal cellulose-amended dog food diet (70% cellulose: 30% dog food) (CADF) (13, 20), and an entirely plant-based diet consisting of granulated root tubers of cassava (*Manihot esculenta*), called Gari purchased from a local African grocery store in Omaha, NE (n = 10 per each treatment). We determined each female’s standard metabolic rate (SMR) by measuring the CO_2_ production twice: on day 1 of the experiment and day 28 after the animals were fed the treatment diet. Before each CO_2_ measurement, all females were starved for 24 hours to ensure they were in a post-adsorptive stage. CO_2_ production by each female was measured using open-flow respirometry (26). Briefly, insects were weighed and placed into a 10cc syringe as a respiration chamber. The syringe was placed into a dark incubator set to 22 °C and connected to the respirometry system for 20 minutes. Cockroaches were acclimated to the 22 °C temperature for ~30 minutes. The 5–7 °C temperature difference between the insect colony and the incubation temperature for the SMR measurement is within the ∼10 °C range within which mass-specific SMR is not significantly affected by temperature changes in cockroaches (27, 28). Air was pushed through the system at a constant rate of 150 mL/min with a mass-flow meter (SS4; Sable Systems, North Las Vegas, NV, USA). CO_2_ and water vapor removed from the air flowing into the insect-containing syringe by passing it through soda lime (Fisher Scientific™, Pittsburgh, PA, USA) and drierite (Drierite Co. Ltd, Xenia, OH, USA), respectively. The air entered the syringe through a rubber stopper in the plunger opening of the syringe barrel and left the syringe through the tip of the syringe. The air containing insect-produced CO_2_ was routed through a condensation column and subsequently through drierite (Drierite Co. Ltd, Xenia, OH, USA) to remove water vapor. This vapor-free air was then moved into the CO_2_ analyzer (Qubit S151, Kingston, Ontario, Canada). CO_2_ production was recorded every second using an electronic interphase (UI-2; Sable Systems, North Las Vegas, NV, USA) and the Expedata software 1.6.0 (Sable systems, North Las Vegas, NV, USA). The baseline was determined before and after each trial by measuring CO_2_ content of the air flowing through the empty syringe for an average of 5 minutes.

We calculated the mass-independent CO_2_ production (VCO_2_) by each insect using the equation: VCO_2_ (mL CO_2_ h^−1^) = flow rate (150 mL/min) × (FeCO_2_ − FiCO_2_)/1 − FeCO_2_ × [1 − (1/RQ)] (26). FiCO_2_ is the CO_2_ content of the air flowing into the syringe, which is 0, and FeCO_2_ is the mean amount of CO_2_ produced during the 20-minute trial by each insect minus the baseline. The baseline is the mean CO_2_ content of the air measured before and after the trial. RQ is the respiratory quotient. We estimated VO_2_ (mL O_2_ h^−1^) as: VO_2_ (mL O_2_ h^−1^) = RQ × VCO_2_ (mL CO_2_ h^−1^). We chose an RQ of 0.85 because the insects had fasted for 24 hours, were postabsorptive, and presumably used mostly fatty acids (29). Furthermore, we included the respiratory (RQ) in the equation to control for errors in the measurement of CO_2_ production (VCO_2_) when oxygen consumption is not simultaneously measured (26). As a conversion factor to express metabolic rate as energy consumption instead of oxygen use, we used 20.8 J mL^−1^ O_2_ (30). We calculated the mass-independent metabolic rate (MR) using the equation: MR (J h^−1^) = VO_2_ (mL O_2_ h^−1^) × 20.8 (J mL^−1^ O_2_). To determine mass-specific MR, we weighed each cockroach immediately before and after measuring CO_2_ production to control for mass change during the CO_2_ measurements. We calculated mass-specific MR by dividing the mass-independent MR by the mean mass.

### DNA extraction, sample processing, and Illumina sequencing

After measuring CO_2_ production on day 28, cockroaches were killed by freezing and storing at −20 °C. Before DNA extraction, we surface-sterilized insects (DF = 5, CADF = 10, Gari = 10) by washing them in a 1% detergent solution for one minute and two one-minute rinses in deionized water. The digestive tract was removed and used for DNA extraction, and the remaining insect carcass (head, thorax, legs, and abdomen) was frozen at −80 °C for stable isotope analyses. According to the manufacturer’s directions, DNA extraction was done using the Qiagen DNeasy Blood & Tissue Kits (Qiagen Inc. Germantown, MD, USA). We verified the presence of the microbial 16S rRNA marker gene in all extracted DNA samples via PCR using the universal 27F and 1492R bacterial primer pair (31). Samples were subsequently submitted for high-throughput paired-end Miseq library preparation and sequencing at the University of Nebraska Medical Center Genomics Core. Briefly, a limited cycle PCR reaction was performed on each sample to create a single amplicon, including the V4 (515-F) and V5 (907-R) variable region (32). The resulting libraries were validated using the Agilent BioAnalyzer 2100 DNA 1000 chip (Agilent, Santa Clara, CA, USA), and DNA was quantified using Qubit 3.0)(Qubit™, Thermofisher, Waltham, MA, USA). A pool of libraries was loaded into the Illumina MiSeq at 10 pM. The pool was spiked with 25% PhiX (a bacteriophage) at 10 pM for MiSeq run quality as an internal control (33) to generate 300 bp paired ends with the 600 cycle kit (version 3). The raw reads were deposited into the Sequence Read Archive database (BioProject Number: PRJNA1017785).

### Mass and SMR data analyses

Mass and mass-specific SMR data were normally distributed. We evaluated the impact of diet on cockroach SMR on day one and day 28 using repeated measures ANOVA in JMP PRO using the Standard Least Squares (SLS) regression approach with a Restricted Maximum Likelihood (REML) estimation method. The model included dietary group (DF, CADF, and GARI) and time (day 1 and day 28) as fixed factors, their interaction, and individual females as random factors. We used an unbounded covariance components structure for the dataset. Data used in the analyses is provided in **Supplementary Table S1**.

### Illumina sequence data processing and analyses

Acquired fastq primer-trimmed Miseq paired-end reads from the sequencing center were processed using DADA2 (34). Reads with more than two expected erroneous base calls were identified as part of the PhiX bacteriophage genome for quality control, and less than 175 base pairs across both forward and reverse reads were filtered out. Forward reads were truncated to 250 base pairs, and reverse reads to 200 base pairs. Truncation was done to maintain median quality scores above 30 across samples. Reads were merged, and chimeras were subsequently filtered out. We determined amplicon sequence variants (ASVs) and representative sequences against the SILVA 138.1 16S rRNA gene reference database (35). We combined the count and taxonomy information for the generated ASVs into a classical OTU/ASV table, and further analyses were carried out in QIIME v.1.8 (36, 37). Before analyses, we curated the table by removing unclassified reads at the bacterial or archaeal domain level and assigned to Eukaryota. Furthermore, for this analysis, we removed all reads assigned to the cockroach obligate fat body endosymbiont, *Blattabacterium* sp. This was done to avoid skewing our analyses of gut bacteria with the endosymbiont since our focus was explicitly on EAA provisioning by the gut bacteria and not the endosymbiont. Finally, samples with less than 1000 reads per sample were removed from the table before analyses. We then summarized the filtered and curated ASV table to the family level, and all subsequent analyses were carried out using this table.

For diversity analyses, we rarefied the family-level table to a minimum acceptable number of 1990 reads per sample across all samples to ensure we had enough replicates per treatment group. Furthermore, given the relatively low number of reads across samples, we performed rarefactions at each level up to 1990 a maximum of ten times. The rationale and justification for rarefying have been discussed elsewhere (38, 39). For alpha diversity, the diversity matrices chao1 (40), Simpson’s index (41), and Shannon’s evenness (42) were calculated in QIIME. Significant differences among categorical groupings were determined using the non-parametric Wilcoxon tests in JMP Pro 15 (S.A.S., Cary, NC, USA). We generated the Bray-Curtis dissimilarity distance matrix (43) using the 1990-rarefied table. The distance matrix was used to calculate non-metric multidimensional scales (NMDS) in QIIME. The NMDS scales are used to visualize categorical sample groupings that differ in microbiome composition. After that, we tested for differences among these categorical groupings via permutational multivariate analysis of variance (PERMANOVA) (44) in QIIME using the Bray-Curtis distance matrix as input. We assessed significant differences in abundance of ASV between dietary groups using the group_significance command in QIIME.

### Essential amino acid *δ^13^C* quantification and provisioning

Before quantifying δ^13^C_EAA_, *Diploptera* carcass samples were lyophilized under vacuum at −80 °C for 48 hrs. Samples were then pulverized and submitted for analysis at the Stable Isotope Facility at the University of California, Davis (Davis, CA, USA). Briefly, samples were first acid-hydrolyzed for 70 min under a N_2_-gas headspace in 6M HCl at 150 °C. Samples were then derivatized as *N*-acetyl methyl esters via methoxy carbonylation-esterification (NACME) (45, 46). Essentially, derivatized samples were injected into a splitless liner at 260 °C and separated on an Agilent DB-35 column (60 m × 0.32 mm ID × 1.5 µm film thickness) at a flow rate of 2 mL/min under the following temperature program: 70 °C (hold 2 min); 140 °C (15 °C/min, hold 4 min); 240 °C (12 °C/min, hold 5 min); and 255 °C (8 °C/min, hold 35 min). Compound-specific isotope ^13^C-amino acid analysis (δ^13^CA) was carried out using a Thermo Trace GC 1310 (GC; Thermo Fisher Scientific, Waltham, MA, USA) coupled to a Delta V Advantage isotope ratio mass spectrometer via the GC IsoLink II (Thermo Electron, Bremen, Germany) (see 46 for analytical details). All samples were analyzed in duplicate. Replicates of the quality control and assurance reference materials are measured every five samples. Standard exogenous carbon addition, kinetic isotope effects from derivatization reagents procedures, and normalization to the international reference for δ^13^C VPDB, as well as calibrated amino acid mixture, UCD AA3, and multiple natural materials were carried out as per facility practices. δ^13^C-EAA data from all samples were obtained for isoleucine, leucine, lysine, phenylalanine, threonine, and valine. The mean standard deviation for all samples was ± 0.21 ‰, well below the established quality control value of ± 0.44%. Final accuracy, as determined by the mean absolute difference in the measured and known δ^13^C values of EAAs from a quality assessment mixture of amino acids, was within ± 0.46 ‰. Analyses of δ^13^C_EAA_ values and enrichment among insect samples and dietary groups (Gari, DF, and CADF) were carried out using ANOVA with insect treatment groups and amino acids (all six EAAs) as factors, following normalization to respective dietary δ^13^C_EAAs_ in JMP (SAS).

To investigate EAA provisioning by gut microbiota, we first examined the δ^13^C offset between consumers and their diets (47). A δ^13^C offset exceeding 2‰ typically signals that the gut microbiota supply EAAs to the host. Without such microbial input, the host would solely depend on its diet for these vital nutrients (3, 4). To pinpoint the origins of *de novo* synthesized EAAs in host tissues, we employed a method known as ^13^C-EAA fingerprinting (5). This technique utilizes linear discriminant function analysis (LDA) and relies on δ^13^C-EAA training data with bacterial, fungal, and plant origins. Our training data was adapted from Larsen et al. (6) following interlab calibrations. Subsequently, we carried out a supervised discriminant analysis to categorize insect and dietary samples into their respective classifier groups (6), followed by a Wilks Lambda test of the accuracy of separation of classifiers. This method also tests for significant differences among centroids of the classifiers. The LDA was executed using the R package MASS (R version 4.2.2.; http://www.R-project.org).

## Results

### Dietary effects on body mass change and SMR

**Figure 1:**
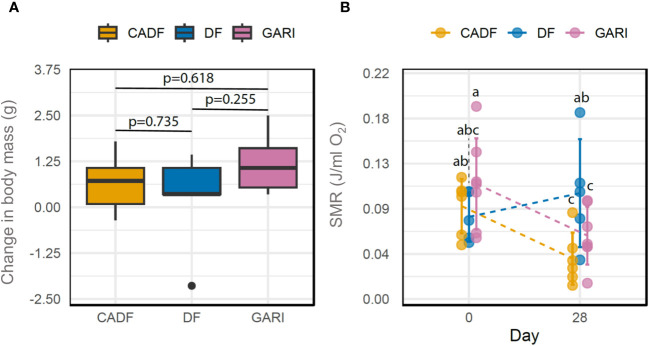
Changes in body mass between day 28 and day 1 (g) **(A)** and Standard metabolic rate (J/ml O_2_) (SMR) **(B)** between day 1 and day 28 in females of *D. punctata* fed on three different diets (DF, CADF, Gari).

The Dietary group × time interaction on body mass change was not significant (*P* = 0.29), and neither was the dietary group (*P* = 0.89), but time (F _(1,14)_ = 7.66, *P* = 0.015) was significant. Overall, average body mass significantly increased over time, ranging from a 3.48% increase in the DF group to an 8.85% increase in the CADF group and a 17.04% increase in the Gari group from day 1 to day 28. This change in body mass between day 1 and 28 on all three diets is represented in **Figure 1A**.

We found a significant Diet × Time interaction on SMR of cockroaches (F-value _(2,15)_ = 3.87; *P* = 0.045), as well as a significant time effect on SMR (F-value _(1,15)_ = 4.76; *P* = 0.045). Mass-specific SMR was constant for DF-fed cockroaches, whereas SMR decreased in CADF-fed and Gari-fed cockroaches by 16.3% and 33%, respectively (**Figure 1B**).

### Dietary effects on the gut microbiome of females

Quality trimming and filtering of the raw data resulted in 3,053,626 combined reads retained from an initial 4,543,250 reads (67%) that were then assigned to 3,580 ASVs. Subsequent curating of the ASV table yielded 3,529 ASVs (mean reads per sample = 35,268; Minimum: 1991, Maximum: 95,875) distributed across 22 insect samples (DF = 5, CADF = 9, Gari = 8), and a total of 775,896 reads. Rarefaction curves indicated that microbial diversity was sufficiently covered across samples.

**Figure 2:**
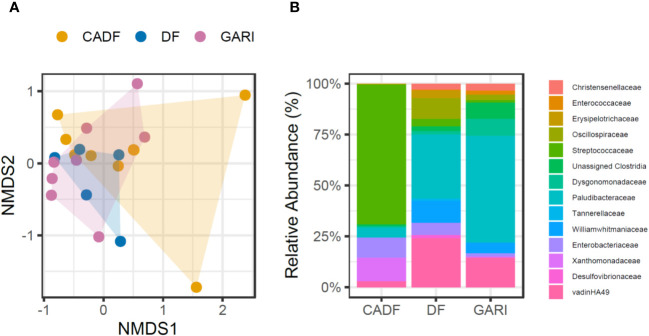
**(A)** Composition of the gut microbiome community in *D. punctata females* fed three diets (DF, CADF, Gari). We did not find significant differences among the dietary groups (PERMANOVA, test statistic = 1.04, P = 0.37; Stress 0.10386). **(B)** Differentially abundant bacterial taxa in the gut microbiome of *D. punctata females* fed different diets (DF, CADF, Gari).

There were no significant differences in gut bacterial species richness estimates across diets (chao1, χ2 = 0.75, *P* = 0.68; Shannon’s index, χ2 = 0.60, *P* = 0.74; Simpson’s index, χ2 = 0.45, *P* = 0.80). We did not find significant differences in microbial community composition of the cockroach gut microbiomes across the three diets (PERMANOVA, test statistic = 1.04, *P* = 0.37) (**Figure 2A**). However, we found differences in the abundance of 20 ASVs belonging to five bacterial phyla (Firmicutes, 47.88%; Bacteroidota, 37.69%; Planctomycetota, 13.27%; Proteobacteria, 0.68%; and Desulfobacterota, 0.45%) and fourteen (14) bacterial families in the gut microbiome of females fed the three diets (*P* < 0.01) (**Figure 2B**) (**Supplementary Table S2**). In the CADF diet group, microbiomes were dominated by Firmicutes (*Streptococcaceae*, 68.7%), Proteobacteria (*Xanthomonadaceae*, 11.7% and *Enterobacteriaceae*, 9.82%). The gut microbiomes of the DF diet group were dominated by Bacteroidota (*Paludibacteraceae*, 31.4%, *Williamwhitmaniaceae*, 10.82%, *Tannerellaceae*, 1.1%), Planctomycetota (vadinHA49, 24.03%), Firmicutes (*Oscillospiraceae*, 10.18%, *Erysipelotrichaceae*, 3.68%, *Christensenellaceae*, 3.04%), and Desulfobacterota (*Desulfovibrionaceae*, 1.52%). Finally, the gut microbiomes of cockroaches fed on Gari were dominated by Bacteroidota (*Paludibacteraceae*, 52.3%, *Dysgonomonadaceae*, 8.2%), Planctomycetota (vadinHA49, 14.43%), and Firmicutes (Unassigned Clostridia, 8.03%, *Christensenellaceae*, 3.5%, and *Enterococcacea*e,1.8%).

### Dietary effects on microbial essential amino acid provisioning

**Figure 3:**
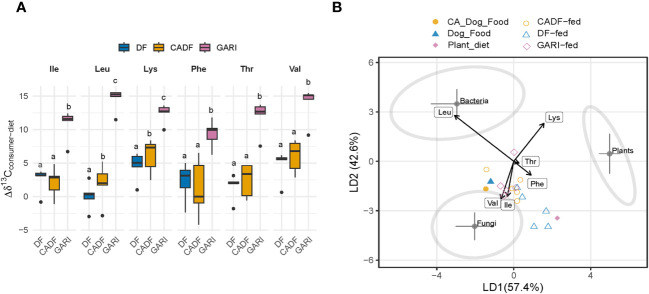
**(A)** ^13^C-offsets (enrichment or depletion) of essential amino acids in diets relative to the respective insect samples. Values represent mean values (± s.e.m.) for duplicate insect and dietary samples. Ile, isoleucine; Leu, leucine; Lys, lysine; Phe, phenylalanine; Thr, threonine; Val, valine. **(B)** A linear discriminant function analysis (LDA) plot based on δ^13^C_EAA_ from DF-fed (N = 5), CADF-fed insects (N = 5), and Gari-fed insects (N = 5), their respective DF (N = 1) and CADF (N = 1) diets, and three classifier groups [fungi (N = 9), bacteria (N = 12) and plants (N = 11)]. The shaded ellipses signify the 95% confidence limits for each classifier group. The essential amino acids used were Ile, isoleucine; Leu, leucine; Lys, lysine; Phe, phenylalanine; Thr, threonine; Val, valine. Our results suggest that diet was not insects’ only source of EAAs.

We obtained δ^13^C_EAA_ data for six EAAs. Our data analysis revealed significant δ^13^C enrichment relative to diets across all six EAAs for all three treatments (F_(5,102)_ = 6.41, *P* < 0.0001). Insects fed on Gari exhibited the highest δ ^13^C-enrichment (4.2 ± 0.4, mean ± S.E. across all EAAs), followed by those on the DF diets (2.6 ± 0.4 across all EAAs). The CADF-fed insects showed the least enrichment (1.8 ± 1.0 across all EAAs) (**Figure 3A**). In terms of individual EAAs in the Gari treatment, Lys had the highest δ ^13^C enrichment (4.2 ± 0.6), followed by Val (3.9 ± 0.6) and Ile (2.6 ± 0.6). The δ^13^C-enrichments in EAAs of sampled insects relative to the three diets are shown in **Figure 3A**. The observed significant ^13^C offsets in these EAAs suggest gut microbial provisioning to the host of these essential nutrients. We investigated the origins of these *de novo* synthesized EAAs in the studied cockroaches using the δ ^13^C-EAA fingerprinting approach. The model based on training data from Larsen et al. (6) successfully separated bacteria (N = 11), fungi (N = 9), and plants (N = 12) into distinct groups (F = 55, *P* < 0.0001; Wilk’s lambda = 0.007, a test of the appropriateness of separation of classifiers and for group membership prediction of non-classifier samples). The supervised LDA with samples with unknown EAA origins (**Figure 3B**) assigned the DF and CADF diets near the fungal classifier group. This can be attributed to the composite nature of the DF and CADF diets. The Gari diet was assigned between the fungi and plant classifier groups. For insect samples, the Gari-fed samples exhibited the most displacement from the dietary source towards bacterial and fungal classifiers, indicating possible fungal and bacterial sources of EAAs. In contrast, the DF-fed and CADF-fed insect samples were close to the fungal classifier group, possibly reflecting the reliance on fungal crude protein in the diet.

## Discussion

Our results offer a framework for investigating how an organism’s gut microbiome mediates various facets of its metabolic phenotype in response to external stressors, such as diet. As anticipated, we detected enhanced gut microbial EAA provisioning in *Diploptera punctata* fed the more homogenous, granulated, and starchy plant-based diet (Gari) relative to those fed the composite diets (DF and CADF). Over 28 days on the respective diets, we did not detect any significant diet-induced changes on the gut microbial community of *D. punctata*. However, despite this lack of significant dietary impact, there were notable shifts in the relative abundances of several bacterial taxa in the gut microbiomes across dietary groups.

Bacterial phyla typically associated with cockroaches and wood-feeding termites, such as Firmicutes, Bacteroidota, and Proteobacteria (48), were detected in high abundances in the gut microbiomes of females in this study. By the end of the feeding period, the gut microbiome of cockroaches fed with CADF mainly comprised bacteria known for breaking down cellulose and recycling nitrogen – *Streptococcaceae, Xanthomonadaceae*, and *Enterobacteriaceae*. These bacteria are commonly found in wood-feeding insects (49–52) and are especially abundant in our wood-feeding cockroaches (mainly, *Streptococcaceae*) (53) (**Figure 2B**). In contrast, gut microbiomes of cockroaches fed Gari were dominated by anaerobic lignocellulolytic bacterial families *Paludibacteraceae* (54, 55), vadinHA49 (56, 57), *Dysgonomonadaceae* (58) and unassigned clostridia (59) (**Figure 2B**). The DF-fed gut microbiomes share some similar bacterial composition to the Gari-fed gut microbiomes, but were further distinguished by the increased abundances of some functionally relevant taxa, such as proteolytic anaerobes *Williamwhitmaniaceae* (60), sulfate-reducing bacteria *Desulfovibrionaceae* (61), anaerobic lipid hydrolyzing bacteria *Erysipelotrichaceae* (62), and amino acid degraders, *Tannerellaceae* (Candidatus Aminobacteroidetes) (63) (**Figure 2B**). The increase in abundances of these families might reflect the high organic-matter content in the guts of the DF-fed cockroaches. In a previous study on *D. punctata* (13), ten bacterial families showed differences in abundance in the gut microbiomes between DF and CADF-fed female gut microbiomes (13), as opposed to 14 bacterial families in this study. Of these taxa, only differences in *Xanthomonadaceae* were detected in both studies. The inclusion of the starchy plant diet may have allowed for the resulting emergence of *Dysgonomonadaceae, Enterococcaceae*, and Unassigned Clostridia in these cockroaches and the loss of some bacterial families in the phylum Firmicutes (*Erysipelotrichaceae*, Unassigned Clostridia, and *Christensenellaceae*) from the Gari-fed gut microbiomes in this study (**Figure 2B**). The distinct bacterial profiles across diets may hint at underlying shifts in gut microbial functions, such as EAA provisioning.

There is extensive evidence for the relevance and importance of insect gut microbial associates in making significant contributions to their insect hosts’ physiology, fitness, and ecology. Insect gut bacteria are postulated to contribute to the digestion of recalcitrant materials provisioning of biological nitrogen (64–68), aiding in detoxification of plant secondary compounds, and providing protection against parasites and predators (69–71). A function of the gut microbiome that has received less attention is the provisioning of EAAs (10–13).

This study expands on prior work (13) by incorporating a Gari diet, a uniform dietary source which is made from ground cassava or Yucca root tubers. The δ^13^C-EAA patterns of the Gari diet fell between those of plants and fungi in the LDA plot. The Gari samples were slightly more ^13^C-enriched across all six EAAs (−24.98 ± 6.36, mean ± std) compared to all the plant classifier samples used in the LDA (−26.25 ± 8.47), and thus fell between those of plants and fungi in the LDA plot. It is possible that the low-protein Gari diet made from tubers may have a different overall δ^13^C-EAA fingerprints than the leaves and stems of the plants used to generate the plant classifier group in the LDA. Though less pronounced, similar differences in δ^13^C-EAA between green leaves and other plant parts such as roots (72, 73) (7) and tubers (74, 75) have been reported. The combination of high starch and low protein content in Yucca tuber may act as a confounding metabolic factor during EAA biosynthesis that could skew their δ^13^C-EAA patterns relative to the training data based on green leaves (76). Thus, further studies using plant green leaves, leafy vegetables, fruits, and possibly fungi as dietary materials are planned to investigate microbial EAA supplementation more definitively in *D. Punctata* in relation to DF and CADF diets. However, irrespective of how the Gari diet is categorized, both the δ^13^C-EAA fingerprints and the insect-to-diet δ^13^C-EAA offset indicate that bacterial symbionts in the Gari-fed insects provide *de novo* synthesized EAAs to their host.

The high dietary protein content in both the DF and CADF diet compared to the Gari diet, made it challenging to assess the extent of gut bacterial EAA provisioning to the DF-fed and CADF-fed insects. This was further compounded by the possibility of uneven gut microbial contributions of *de novo* synthesized EAAs to the host on the DF and CADF diets. EAA provisioning in the DF and CADF insects appeared to be similar despite the CADF having lower overall available protein because it is essentially a diluted DF diet. Previous studies (7, 10) how shown that diets rich in hardly digestible plant fibers lead to higher gut microbial EAA provisioning. Documented compensatory feeding behaviors such as high food consumption on low-protein diets to make up for nutritional imbalances may be at play (77–79). Thus, the different impacts of high-protein and high-starch diets on gut microbial composition and functions, further underscores the need for a nuanced understanding of how various dietary components influence microbial activity across various hosts. While this variability makes definitive assessments challenging, it does not make studying gut microbial EAA supplementation impossible. The fingerprinting approach holds promise for other, more tractable insect systems. For example, two recent studies determined different proportions (and concentrations in nmols) of EAAs in the host (hemocoel and digestive tract) relative to diets (phloem sap and peanuts), which is suggestive of extracellular bacterial EAA supplementation in the hemipteran stinkbugs *Megacopta punctatissima* (80) and *Plautia stali* (81). Utilizing the fingerprinting approach in future research would provide a more definitive and quantitative understanding of the bacterial origins of these EAAs. The success of this approach hinges on using insect systems with well-defined diets – such as strictly phloem sap, wood, or green leaves – for more explicit interpretations. Incorporating appropriate dietary controls into the experimental design becomes crucial when such well-defined dietary materials are unavailable.

We also examined the effects of diet on metabolic responses (SMR) and body mass changes over 27 days. The premise was that shifts in SMR, and consequently body mass, would arise from alterations in microbe-derived nutrient provisioning due to diet-induced changes in gut microbiome composition. Across all three dietary groups, we observed an increase in mean body mass from day 1 to day 28. However, these increases in body mass were not mirrored in the mass-specific SMR responses, which are often complex and yield mixed results. For example, the harvestman, *Pachylus paessleri* (Opiliones) (19), decreased its SMR on a low-quality carbohydrate-rich diet relative to a high-quality protein-rich diet, while other insects like *Spodoptera eridani*a larvae (17), the locust *Locusta migratoria* (82), and the American cockroach *Periplaneta americana* (20) all increased their SMRs on low-quality diets. Interestingly, we observed a decrease in SMR in the CADF-fed group by day 28. This diverges from our previous findings (13), where SMR increased after 28 days on the CADF diet. It is worth noting that the earlier study measured SMR in gravid females and at a higher temperature (30 °C), whereas we used unmated females and measured CO_2_ production at 22 °C. Thus, the differing SMR patterns could be related to physiological constraints unique to gravid females vs. the non-gravid ones in this study. Evidence supports that non-gravid females tend to decrease their SMR when fed the CADF diet (Dr. Agustí Muñoz-Garcia, pers. comm.). Possible other differences may be attributed to differences in the age of the females and SMR changes seasonally in *D. punctata* (Dr. Agustí Muñoz-Garcia, pers. comm.). Similarly, the differences in temperature at which SMR was determined in both studies may also account for different SMR response patterns. Overall, invoking the same logic, one can interpret the depressed SMR of the Gari group in this study by day 28 (although higher than the CADF-fed group), as being impacted by changes in both gut microbiome composition and function.

## Conclusions

We found possible indications of diet-dependent SMR responses across dietary groups in this study. We attribute these SMR responses to diet-induced changes in gut microbial community composition and, subsequently, changes in functions such as the provisioning of microbe-derived metabolites (especially EAAs) for energy generation and biosynthesis. This is evidenced by the EAAs provisioning determined in the Gari-fed samples (and associated gut microbial community changes and SMR), possibly reflecting a difference in high starch and cellulolytic diets. Overall, the results in this study support the hypothesis that insect gut microbiota functions might mediate the metabolic phenotype of insect hosts. The complementary approaches outlined here represent one framework in which *D. punctat*a and its gut microbiome interact to potentially affect the physiological outcome in a diet-dependent manner, with utility for other insect systems.

## Data availability statement

The datasets presented in this study can be found in online repositories. The names of the repository/repositories and accession number(s) can be found in the article/**Supplementary material**.

## Ethics statement

The manuscript presents research on animals that do not require ethical approval for their study.

## Author contributions

PA: Conceptualization, Data curation, Formal analysis, Funding acquisition, Investigation, Methodology, Project administration, Resources, Software, Supervision, Validation, Visualization, Writing – original draft, Writing – review & editing. NP: Data curation, Investigation, Methodology, Validation, Writing – original draft. CR: Data curation, Formal analysis, Investigation, Methodology, Software, Writing – original draft. JR: Methodology, Resources, Writing – review & editing. TL: Conceptualization, Data curation, Formal analysis, Investigation, Methodology, Software, Validation, Writing – original draft, Writing – review & editing.
