# Supplementary material for: Enhanced gut microbiome supplementation of essential amino acids in Diploptera punctata fed low-protein plant-based diet
Source: Front Insect Sci. 2024 Apr 22;4:1396984. doi: 10.3389/finsc.2024.1396984 (PMC11073486; doi:10.3389/finsc.2024.1396984)
Supplement: Supplementary file 2 [file Table_1.docx]

**Table S1**. Body mass (in g.), mass difference, SMR (J mL^-1^ O_2_), and mass specific-SMR (both day 1 and 28) of all the females used in this study.

| **Sample** | **Group** | **Day 0 SMR (J mL^-1^ O_2_)** | **Day 28 SMR (J mL^-1^ O_2_))** | **Day 0 Body mass** | **Day 28 Body mass** | **Mass differences** | **Day 0 Mass-specific SMR (J mL^-1^ O_2_)** | **Day 28 Mass specific SMR (J mL^-1^ O_2_)** |
| --- | --- | --- | --- | --- | --- | --- | --- | --- |
| DF 2 | DF | 0.000649369 | 0.022697739 | 0.1914 | 0.199831076 | 0.008431076 | 0.059983531 | 0.113584633 |
| DF 4 | DF | 0.001646795 | 0.016871806 | 0.1237 | 0.160631076 | 0.036931076 | 0.235370485 | 0.105034508 |
| DF 6 | DF | 0.000999955 | 0.015410178 | 0.1377 | 0.151831076 | 0.014131076 | 0.128389243 | 0.101495549 |
| DF 9 | DF | 0.000703006 | 0.00484433 | 0.118 | 0.126331076 | 0.008331076 | 0.105331716 | 0.038346306 |
| DF 10 | DF | 0.00143187 | 0.033454379 | 0.2413 | 0.183231076 | -0.058068924 | 0.104912803 | 0.182580269 |
| DF 11 | DF | 0.000681318 | 0.019509564 | 0.218 | 0.247431076 | 0.029431076 | 0.055255495 | 0.07884848 |
| GARI1 | GARI | 0.015410617 | 0.014653254 | 0.1365 | 0.153431076 | 0.016931076 | 0.112898289 | 0.095503822 |
| GARI2 | GARI | 0.020172184 | 0.01587594 | 0.1401 | 0.164531076 | 0.024431076 | 0.143984182 | 0.096492042 |
| GARI3 | GARI | 0.021628512 | 0.019510932 | 0.207 | 0.275031076 | 0.068031076 | 0.104485565 | 0.070940826 |
| GARI4 | GARI | 0.028645134 | 0.002761957 | 0.1519 | 0.177831076 | 0.025931076 | 0.188578893 | 0.01553135 |
| GARI5 | GARI | 0.023252832 | 0.012147586 | 0.2024 | 0.237431076 | 0.035031076 | 0.114885534 | 0.051162576 |
| GARI8 | GARI | 0.008315063 | 0.011744217 | 0.1651 | 0.173431076 | 0.008331076 | 0.050363799 | 0.067716913 |
| GARI9 | GARI | 0.01981232 | 0.033823016 | 0.2104 | 0.245431076 | 0.035031076 | 0.094165017 | 0.13781065 |
| GARI10 | GARI | 0.012266352 | 0.012254728 | 0.1899 | 0.238931076 | 0.049031076 | 0.064593743 | 0.051289802 |
| GARI11 | GARI | 0.012933796 | 0.012510538 | 0.2155 | 0.232331076 | 0.016831076 | 0.060017614 | 0.053847888 |
| GARI12 | GARI | 0.008337932 | 0.0154159 | 0.1345 | 0.156431076 | 0.021931076 | 0.061992058 | 0.098547556 |
| CADF1 | CADF | 0.011728475 | 0.031747881 | 0.1106 | 0.116331076 | 0.005731076 | 0.106044076 | 0.272909712 |
| CADF2 | CADF | 0.02594788 | 0.016719166 | 0.2177 | 0.208431076 | -0.009268924 | 0.119190996 | 0.080214362 |
| CADF3 | CADF | 0.01511866 | 0.051714091 | 0.2619 | 0.240931076 | -0.020968924 | 0.05772684 | 0.214642676 |
| CADF4 | CADF | 0.016553784 | 0.01367331 | 0.158 | 0.161531076 | 0.003531076 | 0.104770783 | 0.084648173 |
| CADF5 | CADF | 0.026857787 | 0.014756068 | 0.2673 | 0.295931076 | 0.028631076 | 0.100478065 | 0.049863193 |
| CADF6 | CADF | 0.018022816 | 0.006311167 | 0.1448 | 0.167431076 | 0.022631076 | 0.124466965 | 0.03769412 |
| CADF7 | CADF | 0.012548511 | 0.022621599 | 0.179 | 0.228331076 | 0.049331076 | 0.070103415 | 0.099073673 |
| CADF8 | CADF | 0.006693581 | 0.005317534 | 0.1262 | 0.175331076 | 0.049131076 | 0.053039472 | 0.030328529 |
| CADF9 | CADF | 0.008280008 | 0.011514671 | 0.151 | 0.162431076 | 0.011431076 | 0.054834488 | 0.070889583 |
| CADF10 | CADF | 0.030084501 | 0.030050144 | 0.3004 | 0.319031076 | 0.018631076 | 0.100148139 | 0.094191901 |

**Table S2**: Differential abundances of bacterial families significantly varied among the three *Diploptera punctata* dietary treatment groups, DF-fed, CADF-fed, and Gari-fed.

| **Phylum** | **Family** | **Test-Statistic** | **P** | **DF** | **CDF** | **Gari** |
| --- | --- | --- | --- | --- | --- | --- |
| Firmicutes | *Streptococcaceae* | 13.82 | 0.00 | 3.40 | 106.44 | 1.25 |
| Firmicutes | *Unassigned Clostridia* | 11.22 | 0.00 | 1.40 | 0.00 | 0.00 |
| Proteobacteria | *Xanthomonadaceae* | 10.31 | 0.01 | 0.00 | 18.11 | 0.63 |
| Planctomycetota | *vadinHA49* | 10.22 | 0.01 | 22.20 | 4.33 | 14.38 |
| Firmicutes | *Christensenellaceae* | 9.83 | 0.01 | 0.60 | 0.00 | 1.50 |
| Bacteroidota | *Dysgonomonadaceae* | 8.89 | 0.01 | 1.60 | 0.89 | 8.25 |
| Firmicutes | *Oscillospiraceae* | 7.91 | 0.02 | 3.00 | 0.00 | 0.13 |
| Firmicutes | *Christensenellaceae* | 7.66 | 0.02 | 0.80 | 0.11 | 2.00 |
| Firmicutes | *Oscillospiraceae* | 7.15 | 0.03 | 6.40 | 0.67 | 2.50 |
| Proteobacteria | *Enterobacteriaceae* | 7.15 | 0.03 | 5.60 | 15.22 | 1.50 |
| Bacteroidota | *Tannerellaceae* | 7.12 | 0.03 | 1.00 | 0.00 | 0.00 |
| Desulfobacterota | *Desulfovibrionaceae* | 7.12 | 0.03 | 1.40 | 0.00 | 0.00 |
| Firmicutes | *Erysipelotrichaceae* | 7.12 | 0.03 | 3.40 | 0.00 | 0.00 |
| Firmicutes | *Unassigned Clostridia* | 7.12 | 0.03 | 0.80 | 0.00 | 0.00 |
| Firmicutes | *Christensenellaceae* | 7.12 | 0.03 | 0.80 | 0.00 | 0.00 |
| Firmicutes | *Christensenellaceae* | 7.12 | 0.03 | 0.60 | 0.00 | 0.00 |
| Bacteroidota | *Williamwhitmaniaceae* | 6.80 | 0.03 | 10.00 | 0.00 | 5.25 |
| Bacteroidota | *Paludibacteraceae* | 6.68 | 0.04 | 29.00 | 7.78 | 52.38 |
| Firmicutes | *Enterococcaceae* | 6.30 | 0.04 | 0.40 | 0.11 | 1.88 |
| Firmicutes | *Unassigned Clostridia* | 6.15 | 0.05 | 0.00 | 1.33 | 8.00 |
